# Regular consumption of pickled vegetables and fermented bean curd reduces the risk of diabetes: a prospective cohort study

**DOI:** 10.3389/fpubh.2023.1155989

**Published:** 2023-04-27

**Authors:** Yulan Cai, Xiaoxia Yang, Siju Chen, Kunming Tian, Suowen Xu, Renli Deng, Min Chen, Yan Yang, Tao Liu

**Affiliations:** ^1^Department of Endocrinology and Metabolism, The Second Affiliated Hospital of Zunyi Medical University, Zunyi, China; ^2^Department of Endocrinology and Metabolism, Affiliated Hospital of Zunyi Medical University, Zunyi, Guizhou, China; ^3^Department of Preventive Medicine, School of Public Health, Zunyi Medical University, Zunyi, Guizhou, China; ^4^Department of Endocrinology, The First Affiliated Hospital of USTC, Division of Life Sciences and Medicine, University of Science and Technology of China, Hefei, China; ^5^Department of Nursing, Affiliated Hospital of Zunyi Medical University, Zunyi, China; ^6^Department of Chronic Disease Prevention and Control, Guizhou Disease Prevention and Control, Guiyang, Guizhou, China

**Keywords:** diabetes, pickled vegetables, fermented bean curds, prospective, cohort study

## Abstract

**Objective:**

The global incidence of diabetes is rising, in part due to the widespread adoption of poor dietary habits. Fermented vegetables have numerous health benefits and are generally affordable. Here, we examined whether regular consumption of pickled vegetables or fermented bean curd reduces the risk of diabetes.

**Methods:**

A total of 9,280 adults (≥18 years of age) were recruited via multi-stage sampling from 48 townships in China between 2010 and 2012 for this 10-year prospective study. In addition to demographic information, monthly consumption levels of pickled vegetables and fermented bean curd were recorded. Participants were then monitored for diabetes onset. After the final follow-up, logistic regression analyses with multiple covariant corrections were conducted to estimate the changes in diabetes risk associated with consumption of pickled vegetables and fermented bean curd compared to non-consumption.

**Results:**

A total of 6,640 subjects without diabetes at the start of the study were followed up for a median period of 6.49 years, among whom 714 were diagnosed with diabetes during the study. According to a regression model with multivariable adjustment, diabetes risk was significantly reduced by consumption of 0–0.5 kg/month of pickled vegetables (OR = 0.77, 95% CI: 0.63, 0.94) and further reduced by consumption of >0.5 kg/month of pickled vegetables (OR = 0.37, 95% CI: 0.23, 0.60) compared to no consumption (both *P*-trend < 0.001). Consumption of fermented bean curd also reduced diabetes risk (OR = 0.68, 95% CI: 0.55, 0.84).

**Conclusion:**

Regular consumption of pickled vegetables and/or fermented bean curd can reduce the long-term risk of diabetes.

## Introduction

Diabetes is a leading cause of death and disability worldwide (1), mainly due to its associated complications, such as cardiovascular and renal diseases, retinopathy, and other neuropathies. Currently, there are 537 million diagnosed cases of diabetes among the global adult population, and these patients collectively impose a major burden on healthcare systems and national economies (2). The prevalence of diabetes in China has more than quadrupled over the past two decades (3). Diabetes onset and progression are strongly associated with poor diet and lifestyle choices (4); thus, the promotion of effective dietary interventions is among the most promising and cost-effective strategies for reducing the incidence of diabetes and managing existing disease.

Pickling and fermentation are effective methods of preserving foods and can even improve the food's nutritional value. Indeed, the health benefits of these products are well-known (5). It is widely believed that these benefits stem in part from the complex microbial reactions involved in fermentation (6, 7), which create additional nutrients and influence the composition of the gut microbiome. In China, pickled vegetables and fermented bean curd are widely consumed, and recent investigations have shown that regular consumption of these can help to improve poor digestion, lower cholesterol, and prevent gut-borne infections (8). Moreover, the microbiome isolated from pickled foods has potential probiotic properties that can prevent hyperuricemia and intestinal inflammation (9, 10). While probiotic supplements are still not widely accepted in China, most Chinese citizens eat pickled foods. Therefore, promoting the consumption of pickled vegetables and fermented bean curd may be a feasible strategy to enhance the use of probiotics among Chinese citizens and potentially to prevent diseases associated with gut dysbiosis, including diabetes.

However, no studies have yet examined whether the consumption of pickled vegetables and fermented bean curd can reduce the risk of diabetes. We therefore conducted a large-scale, long-term prospective study comparing diabetes onset rates among Chinese adults reporting pickled vegetable or fermented bean curd consumption and non-consumers.

## Methods

### Study participants

**Figure 1:**
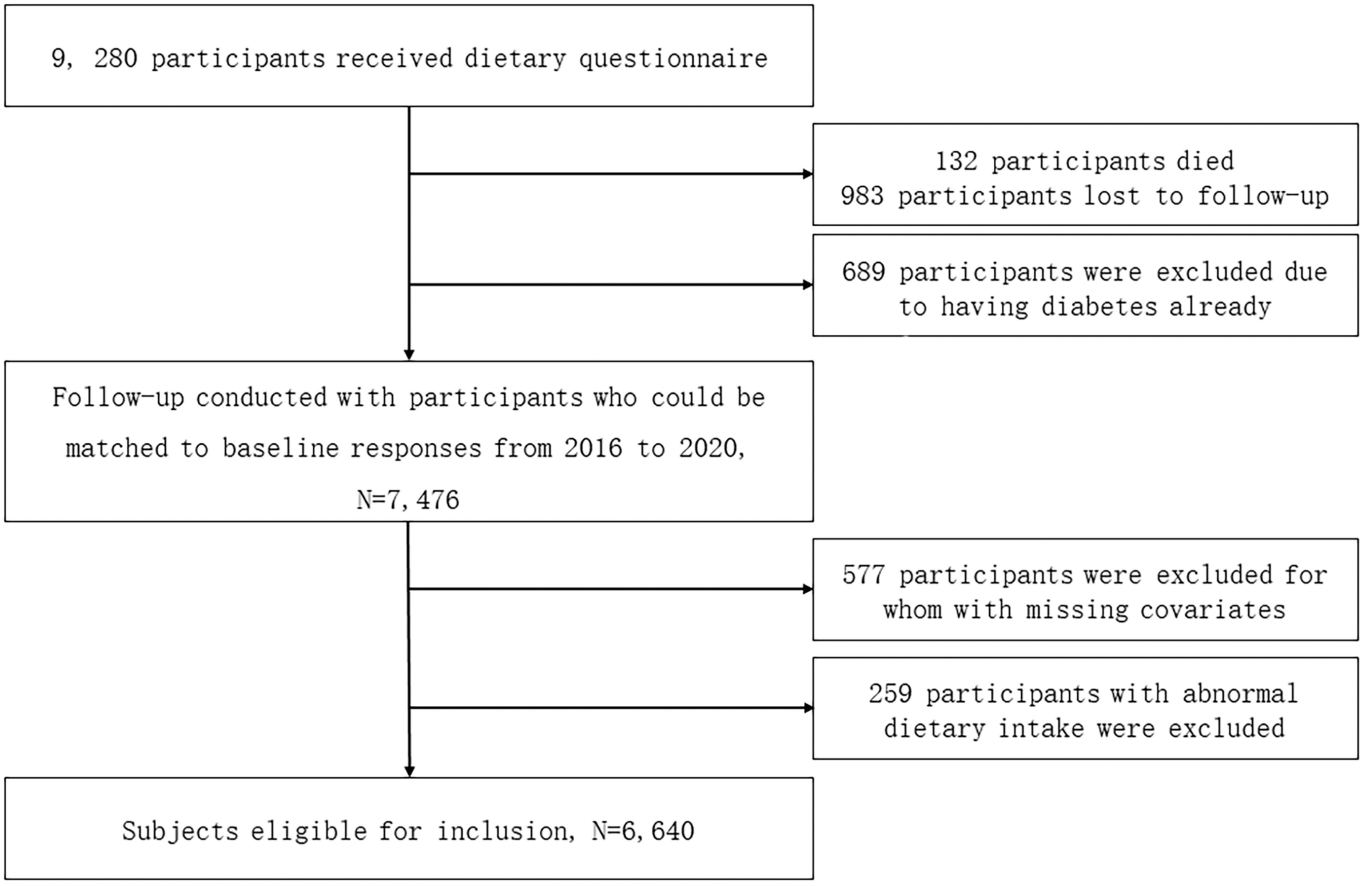
Flow chart showing participant recruitment and data collection. Of the 9,280 participants recruited, 689 were excluded due to having diabetes at baseline. In addition, 983 participants were lost to follow-up, 132 died, 577 were excluded due to missing covariable data, and 259 were excluded for abnormal dietary practices. Therefore, analyses were conducted on data from 6,640 participants.

This was a community-based, prospective cohort study investigating the association of fermented bean curd and pickled vegetable consumption with diabetes incidence. Between 2010 and 2012, a total of 9,280 participants were recruited, *via* multi-stage sampling, from 48 townships throughout various regions of Guizhou Province, China. The inclusion criteria were as follows: (1) age ≥18 years, (2) complete physical and dietary records at baseline and follow-up, and (3) willing to complete the required interviews and questionnaires. Of the initial cohort, 689 were excluded due to having diabetes at baseline, 983 were lost to follow-up, 132 died, 577 were excluded due to missing covariable data, and 259 were excluded due to an abnormal diet. An abnormal diet was defined as a level of consumption of pickled vegetables or fermented bean curd falling outside the interquartile range (as indicated on a box plot) by more than 1.5 times that range when the variable was treated as continuous. After these exclusions, 6,640 participants were included in the final analyses (Figure 1).

This study was approved by the Institutional Review Committee of the Guizhou Center for Disease Control and Prevention (Grant No., 20081010593, S2017-02), and all participants provided written informed consent.

### Baseline data collection

Baseline information, including sociodemographic and lifestyle variables, monthly consumption of pickled vegetables, monthly consumption of fermented bean curd, and history of chronic disease, was collected systemically by well-trained interviewers using structured questionnaires. Trained nurses measured participants' body weight, height, and blood pressure according to a standard protocol (11). Participants were required to fast for at least 8 h before visits to local clinics in the morning for glucose tolerance testing and blood lipid analysis. For oral glucose tolerance testing, venous blood was collected at 0 and 2 h after oral consumption of 75 g anhydrous glucose. Glucose oxidase or hexokinase was used to measure blood glucose concentration under strict quality control procedures. Total serum cholesterol, triglycerides, low-density lipoprotein cholesterol, and high-density lipoprotein cholesterol were measured using standard analytic procedures.

### Pickled vegetable and fermented bean curd intake

Intake rates of pickled vegetables and fermented bean curd were recorded for all participants in kg per month. Data were elicited using the following questions: “Do you eat pickled vegetables?”, “How often do you eat pickled vegetables?”, “How many kilograms of pickled vegetables do you eat per month?”, “Do you eat fermented bean curd?”, and “How many kilograms of fermented bean curd do you eat per month?”. The response options for binary questions were “never” or “yes.”

### Diagnostic criteria for diabetes, hypertension, and dyslipidemia

Diabetes was diagnosed according to the World Health Organization (WHO, 1999) criteria: (1) random plasma glucose ≥200 mg/dl (≥11.1 mmol/L) plus the classic symptoms of thirst, hyperphagia, excessive urination, and unexplained weight loss; or (2) fasting plasma glucose ≥126 mg/dl (≥7.0 mmol/L) and oral glucose tolerance test 2-h glucose ≥200 mg/dL (≥11.1 mmol/L) (12). Hypertension was diagnosed based on three systolic blood pressure (SBP) and diastolic blood pressure (DBP) measurements from the right brachial artery using an electronic sphygmomanometer. Each measurement was collected on a different day, with the participant in a quiet resting state. Participants were prohibited from strenuous exercise within 12 h of the test. Hypertension was defined as blood pressure above 140/90 mmHg on all three measurements (13). Finally, dyslipidemia was diagnosed if triglyceride level was ≥2.26 mmol/L, total cholesterol was ≥6.22 mmol/L, high-density lipoprotein cholesterol was <1.04 mmol/L, and/or low-density lipoprotein cholesterol was ≥4.14 mmol/L (14).

### Assessment of covariates

Trained healthcare workers collected the following pieces of baseline information during face-to-face interviews: (1) sociodemographic factors: sex (male or female), age (18–44, 45–59, or ≥60 years old), area (urban or rural), BMI (kg/cm^2^), education (no formal school, primary or middle school, high school, college/university, or higher), and marital status (single/never married, married, divorced, or other); (2) lifestyle factors: alcohol intake (never, 1-3 days/month, 1-6 days/week, or everyday), smoking (never, sometimes, or always), exercise (never, 1 or 2 days/week, or ≥3 days/week), daily meat intake (g/day), daily vegetable intake (g/day), and daily rice intake (g/day). Information was also collected on disease history, including hypertension (yes/no) and dyslipidemia (yes/no).

### Follow-up and outcome assessment

All participants were invited for face-to-face follow-up interviews during the period 2016–2020. Staff used the same standard questionnaires to assess lifestyle factors and diet as at baseline. Similarly, blood pressure was measured utilizing the same rigorous protocol as at baseline. An oral glucose tolerance test was then performed with blood sampling and analysis as described for baseline measures.

### Statistical methods

Baseline characteristics are expressed in the form of a median (interquartile range, IQR) or *n* (%), and the diabetic and non-diabetic groups were compared on these characteristics *viaχ*^2^ test or Kruskal–Wallis test as indicated. Schoenfeld residual plots revealed violations of the proportional hazard assumptions, so we employed logistic regression analysis to estimate adjusted odds ratios (ORs) and 95% confidence intervals (CIs) for the association between pickled vegetable/fermented bean curd consumption and risk of diabetes. Three separate models were constructed with adjustments for different sets of covariates to confirm the robustness of the associations. Model 1 was a crude unadjusted model; Model 2 was adjusted for age, sex, area, education, and marital status; and Model 3 was further adjusted for exercise, alcohol intake, BMI, smoking, vegetable intake, meat intake, rice intake, hypertension, and dyslipidemia. Trend tests were conducted for each model to evaluate the associations of pickled vegetable consumption and bean curd consumption with diabetes risk at the median value of each covariate. Two sensitivity analyses were then conducted to confirm the associations. First, we excluded individuals diagnosed with diabetes during the first 2 years of follow-up. Second, subjects with abnormal dietary intake were excluded to reduce the potential for reverse causality.

A stratified analysis was also performed. Multipliers were added to the logistic regression model to test for interactions between diabetes-relevant variables and consumption rates of pickled vegetables and fermented bean curd. Odds ratios were then calculated for sex, age, area, education, alcohol intake, smoking, and exercise. Finally, restricted cubic splines were used to explore the potential dose–response pattern, with three knots (25%, 50%, and 75%) selected to smooth the curve.

All data analyses were conducted using SPSS (version 25.0. IBM Corporation, NY, USA) and R (version 4.0.5, R Foundation for Statistical Computing). (Two-tailed) *P* < 0.05 was considered to represent statistical significance in all tests.

## Results

### Differences in demographics between diabetic and non-diabetic groups

Of the 9,260 subjects enrolled, 6,640 were included in the final analyses, with a median follow-up period of 6.49 years (Figure 1). The baseline characteristics of participants divided according to diabetes status at final follow-up are presented in Table 1. Participants diagnosed with diabetes during the study period or at final follow-up were more likely than non-diabetics to fall into the 45–59 years or the 60 years age bracket. The diabetic subgroup also included a greater proportion of subjects with little or no formal education and a lower proportion with a college education. Diabetic participants were slightly more likely to live in rural areas, to smoke occasionally or often, to drink alcohol often, and to have hypertension. Diabetics ate less meat and a significantly smaller proportion consumed pickled vegetables or fermented bean curd.

**Table 1:** Sociodemographic characteristics and consumption rates of pickled vegetables and fermented bean curd among study participants.

**Characteristic**	**Non-diabetic group**	**Diabetic group**	***P*-value**
*N* (%)	5,926 (89.2)	714 (10.8)	
**Sex (%)**			
Male	2,767 (46.7)	344 (48.2)	0.476
Female	3,159 (53.3)	370 (51.8)	
**Age (%)**			
18–44	3,319 (56.0)	319 (44.7)	<0.001
45–59	1,732 (29.2)	239 (33.5)	
≥60	875 (14.8)	156 (21.8)	
**Education (%)**			
No formal school	2,123 (35.8)	308 (43.1)	<0.001
Primary or middle school	3,037 (51.2)	331 (46.4)	
High school	506 (8.5)	59 (8.3)	
College/university or higher	260 (4.4)	16 (2.2)	
**Area (%)**			
Urban	1,922 (32.4)	197 (27.6)	0.01
Rural	4,004 (67.6)	517 (72.4)	
**Marital status (%)**			
Single/never married	576 (9.7)	46 (6.4)	0.001
Married	4,810 (81.2)	576 (80.7)	
Divorce	512 (8.6)	88 (12.3)	
Other	28 (0.5)	4 (0.6)	
**Smoking (%)**			
Always	1,405 (23.7)	207 (29.0)	0.008
Sometimes	461 (7.8)	52 (7.3)	
Never	4,060 (68.5)	455 (63.7)	
BMI (median [IQR])	22.24 [20.45, 24.47]	22.97 [20.71, 25.75]	<0.001
**Alcohol intake (%)**			
Always	407 (6.9)	69 (9.7)	0.006
1–6 days/week	687 (11.6)	93 (13.0)	
1–3 days/month	494 (8.3)	43 (6.0)	
Never	4,338 (73.2)	509 (71.3)	
**Exercise (%)**			
Never	5,447 (91.9)	666 (93.3)	0.076
1 or 2 days/week	135 (2.3)	7 (1.0)	
≥3 days/week	344 (5.8)	41 (5.7)	
Vegetable intake (median [IQR]) g/day	300.00 (200.00, 500.00)	300.00 (200.00, 500.00)	0.17
Meat intake (median [IQR]) g/day	70.43 (31.90, 117.62)	62.86 (27.74, 110.22)	0.03
Rice intake (median [IQR]) g/day	400.00 (300.00, 500.00)	400.00 (300.00, 450.00)	0.066
**Hypertension (%)**			
No	4,536 (76.5)	504 (70.6)	0.001
Yes	1,390 (23.5)	210 (29.4)	
**Dyslipidemia (%)**			
No	5,827 (98.3)	695 (97.3)	0.081
Yes	99 (1.7)	19 (2.7)	
**Pickled vegetable intake (%)**			
0 kg/month	4,182 (70.6)	558 (78.2)	<0.001
0–0.5 kg/month	1,369 (23.1)	138 (19.3)	
>0.5 kg/month	375 (6.3)	18 (2.5)	
**Fermented bean curd intake**			
No	4,555 (76.9)	595 (83.3)	<0.001
Yes	1,371(23.1)	119 (16.7)	

### Association of pickled food intake with incidence of diabetes

**Figure 2:**
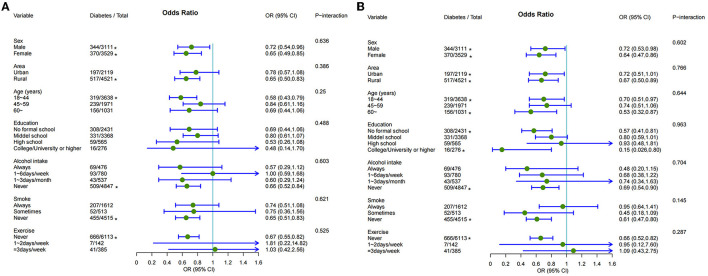
Adjusted odds ratios (ORs) with 95% confidence intervals (CIs) for the associations of pickled vegetable intake **(A)** and fermented bean curd intake **(B)** with diabetes in the indicated subgroups.

A total of 714 participants were diagnosed with diabetes during the median 6.49-year period of follow-up. Table 2 presents the associations of pickled vegetable consumption and fermented bean curd consumption levels with diabetes risk according to three logistic regression models: an unadjusted raw model, and two models adjusted for the variables listed in the methods section. Compared to participants reporting no pickled vegetable consumption (0 kg/month), the risk of diabetes was reduced among participants consuming 0–0.5 kg/month (OR = 0.77, 95% CI: 0.63, 0.94) and further reduced among participants consuming more than 0.5 kg/month (OR = 0.37, 95% CI: 0.23, 0.60; *P*-trend <0.001) according to the full multivariable-adjusted model. Similarly, consumption of fermented bean curd (yes vs. no) was associated with reduced risk according to the fully adjusted model (OR = 0.68, 95% CI: 0.55, 0.84). Consumption of both pickled vegetables and fermented bean curd also reduced diabetes risk according to an adjusted regression model with continuous variables (pickled vegetables: OR = 0.39, 95% CI: 0.26, 0.60, *P* < 0.001; fermented bean curd: OR = 0.17, 95% CI: 0.08, 0.36, *P* < 0.001; Supplementary Table 1). In addition, we used restricted cubic splines to examine the dose-dependence of these effects; we found that the ORs were significantly reduced as pickled vegetable intake increased above 0.318 kg/month and as fermented bean curd intake increased above 0.242 kg/month (Figures 2A, B).

**Table 2:** Adjusted odds ratios for associations of diabetes and pickled vegetable/fermented bean curd intake (treated as categorical variables).

**Classified variable**	**Odds ratio (95% CI)**		
	**Model 1**	**Model 2**	**Model 3**
**Pickled vegetable intake(kg/month)**			
0	1 [reference]	1 [reference]	1 [reference]
0–0.5	0.76 (0.62, 0.92)	0.79 (0.65, 0.97)	0.77 (0.63, 0.94)
>0.5	0.36 (0.22, 0.58)	0.39 (0.24, 0.63)	0.37 (0.23, 0.60)
*P*-trend		<0.001	
**Fermented bean curd intake(kg/month)**			
No	1 [reference]	1 [reference]	1 [reference]
Yes	0.66 (0.54, 0.82)	0.70 (0.57, 0.86)	0.68 (0.55, 0.84)

Model 1: Unadjusted.

Model 2: Adjusted for age, sex, area, education, and marital status.

Model 3: Adjusted for Model 2 + exercise, alcohol intake, BMI, smoking, vegetable intake, meat intake, rice intake, hypertension, and dyslipidemia.

### Predictive value of pickled vegetable intake and fermented bean curd intake and incidence of diabetes in subgroups

**Figure 3:**
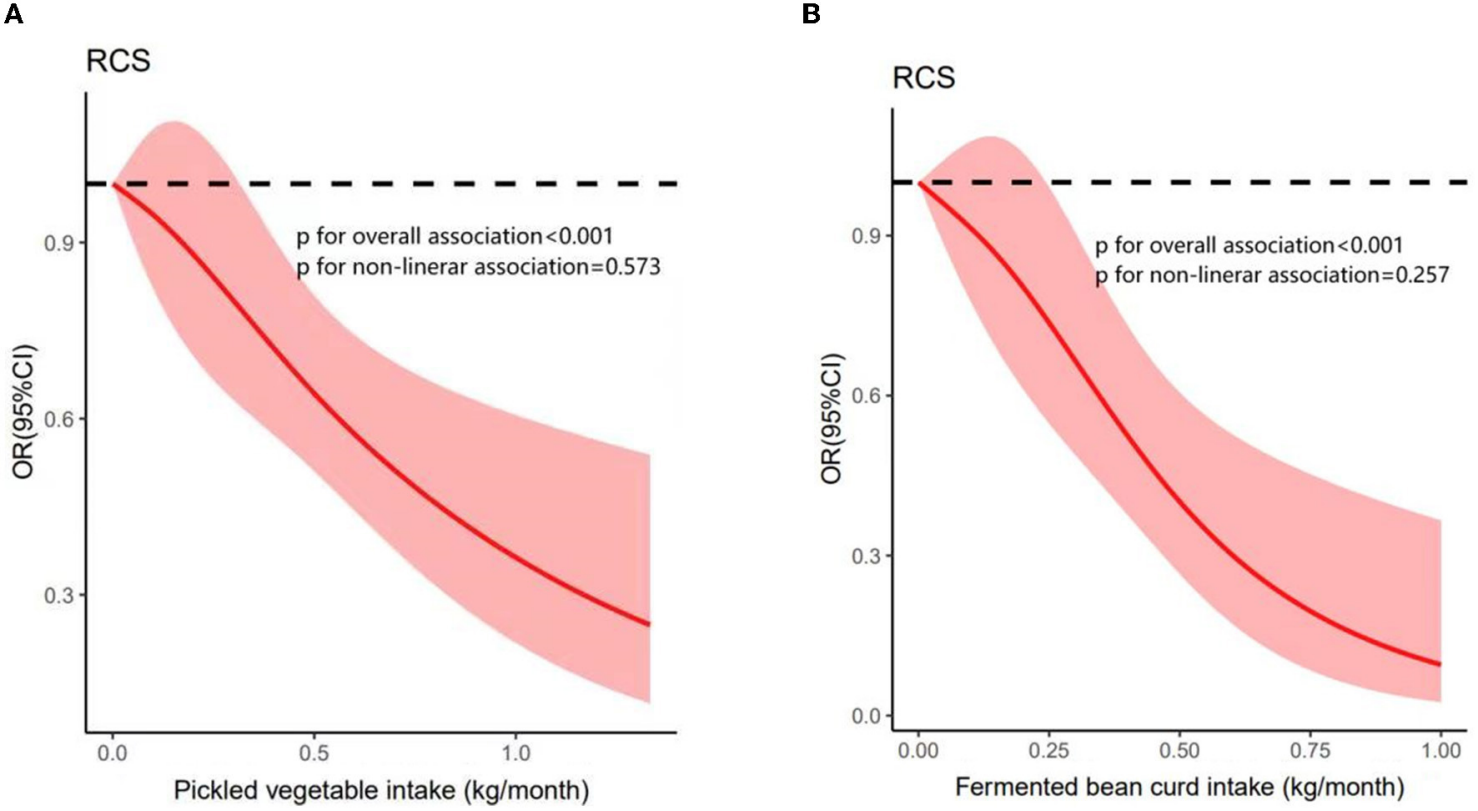
Adjusted ORs (solid line) and 95% Cls (pink shaded area) for the associations of **(A)** pickled vegetable intake and **(B)** fermented bean curd intake with diabetes. The red line represents the estimated OR of diabetes and the pink shaded area represents the 95% Cl around this. The dashed line represents the OR = 1.

As shown in Figures 3A, B, intake of pickled vegetables reduced the risk of diabetes among women, those living in rural areas, participants aged 18–44 years, non-drinkers, non-smokers, and those who never exercised, while consumption of fermented bean curd reduced the risk among women, inhabitants of rural areas, participants older than 60 years, those with a college/university education or higher degree, non-drinkers, non-smokers, and participants who never exercised. To test the robustness of these associations, we conducted several additional sensitivity analyses. We also re-analyzed the results after removing participants diagnosed with diabetes within the first 2 years of follow-up. According to the models including all diabetes cases, diabetes risk was reduced by consumption of 0–0.5 kg/month pickled vegetables (OR = 0.75, 95%: CI 0.61, 0.93) and reduced further by intake of >0.5 kg/month pickled vegetables (OR = 0.35, 95% CI: 0.22, 0.59; *P*-trend <0.001). Similarly, intake of fermented bean curd reduced diabetes risk (OR = 0.67, 95% CI: 0.54, 0.83; Supplementary Table 2). Moreover, these associations were maintained in models including participants with an abnormal diet and regardless of whether consumption level was treated as a continuous or a categorical variable (Supplemental Tables 3, 4).

## Discussion

In this study population, regular consumption of pickled vegetables or fermented bean curd was associated with a lower risk of diabetes onset during follow-up, even after controlling for multiple diabetes-related demographic and clinical covariates. Therefore, dietary supplementation with these accessible food products may contribute to diabetes prevention and management.

The global prevalence of diabetes has increased dramatically in the past decade, primarily due to high-energy diets and sedentary lifestyles (15). In the present study, logistic regression analysis revealed a 23% reduction in diabetes risk among participants consuming 0–0.5 kg/month of pickled vegetables and a 63% reduction among those consuming more than 0.5 kg/month compared to non-consumers. In addition, the consumption of fermented bean curd reduced diabetes risk by 32%. Significant risk reductions were observed at a pickled vegetable consumption level of just 0.318 kg/month (or higher) and at a fermented bean curd consumption level of 0.242 kg/month or higher, despite the fact that these products may have deleterious effects on blood pressure control due to their high salt content. It has been documented that patients with hypertension exhibit reduced abundance of *Bifidobacteria* in the gut (16). Pickled foods improve gut health and reduce blood pressure by supplementing probiotics, including *Bifidobacteria*. Pickled vegetables also contain alpha-linolenic acid, 5-hydroxymethylfurfural, and sitosterol, which act as anti-atherosclerotic, cardiovascular disease-preventing, anti-inflammatory, and lipid-lowering agents (17). Nonetheless, large-scale prospective cohort studies are needed to examine the precise relationships among pickled vegetable consumption levels, total salt intake, and hypertension.

There were also substantial differences in demographic characteristics between the diabetic and non-diabetic subgroups. While the gender ratio did not differ between participants who remained non-diabetic and those who were diagnosed with diabetes during follow-up, a higher proportion of those who were diagnosed with diabetes by the end of the study were older than 45 years, lacking in formal education, living in rural areas, habitual smokers, and regular alcohol drinkers. Those diagnosed with diabetes were also slightly but significantly heavier. Similarly, in the United States, diabetes rates are higher in rural areas than in urban areas (18). Previous studies have also demonstrated that smoking increases the risk of prediabetes and diabetes in the general population (19). In addition, alcohol abuse is associated with an increased risk of diabetes, while low-to-moderate consumption of wine may have beneficial effects (20). Each of these unhealthy lifestyle factors (excessive body weight, smoking, and excessive drinking) contributes moderately to diabetes risk, suggesting that multiple lifestyle changes are required to substantially reduce the risk (21).

Aberrant changes in gut microbiome composition (termed dysbiosis) increase diabetes risk by enhancing insulin resistance, which accounts for ~90% of all diabetes cases worldwide (22–25). Multiple lines of evidence suggest that dietary probiotics able to restore normal gut microbiome composition can help to prevent and mitigate metabolic diseases, including diabetes, by improving the integrity of the intestinal barrier, reducing gut inflammation, and maintaining insulin sensitivity (26–28). These probiotic effects stem from the unique characteristics of fermentation (29). Pickled food is a natural carrier of probiotics with direct antagonistic effects against pathogenic gut microbes. Indeed, probiotics are widely used to treat or prevent infectious diarrhea, irritable bowel syndrome, inflammatory bowel disease, *Helicobacter pylori* infection, and lactose intolerance (30, 31). Various probiotics have been isolated from pickled vegetables and fermented bean curd products. Studies have reported that *Lactobacillus* and *Weissella* are present in pickled food, such as pickled vegetables and soybean paste (32, 33). *Weissella* is the dominant bacterium in the early fermentation process and is partially replaced by *Lactobacillus* in the late fermentation process (34), a shift that contributes to flavor formation (35, 36). The initial presence of *Weissella* not only improves the flavor of pickled foods but also inhibits the growth of pathogenic microbes by producing acid and alcohol, thereby ensuring food safety (37, 38). Some pickled food preparation methods use *Lactobacillus plantarum* as a starter, which not only maintains the activity of acid-fast bacteria but also survives in the gastrointestinal tract and inhibits the growth of pathogenic *Candida albicans* (39). Indeed, strains of *Lactobacillus brevis* DF01 and *Pediococcus acidilactici* K10 isolated from pickled vegetables show stronger probiotic activity than strains isolated from animals (40). Overall, fermented food has demonstrated beneficial effects due to its probiotic-like function, suggesting potential therapeutic applications for diabetes (41).

The long history of pickled food consumption confirms the safety of the microorganisms used for fermentation, and more recent studies have reported additional health benefits. Thus, Chinese pickled food is suitable for daily consumption as a probiotic supplement. This study shows that appropriate intake of probiotic-rich pickled foods is an efficacious and cost-effective strategy for reducing the risk of diabetes.

### Strengths and limitations

Strengths of this study include the large representative sample size, prospective cohort design, detailed classification, and adjustment of potential covariant risk factors, which collectively yielded statistical strength sufficient to reveal that consumption of pickled vegetables or fermented bean curd can reduce diabetes risk. Furthermore, these associations were confirmed by multiple sensitivity analyses. First, we excluded patients diagnosed with diabetes during the first 2 years of follow-up. Second, we constructed three models that adjusted for different sets of confounders. Third, we explored the individual interactive effects of sex, type of area of residence, age, education, alcohol intake, smoking, and exercise on the associations of pickled vegetable consumption and fermented bean curd consumption with diabetes.

Nonetheless, these results should be interpreted with caution. Our study did not evaluate the precise details of pickled food intake, such as frequency per week, specific type consumed, and changes in consumption over time. However, monthly intake provides a better estimation of regular total intake, which in turn is more likely than short-term intake to influence diabetes-related factors like gut microbiome status. Furthermore, we did not collect data on intestinal microbiome composition to confirm any mediating effect of this on diabetes risk.

In summary, this large prospective analysis provides evidence that the consumption of pickled vegetables and fermented bean curd lowers the risk of diabetes. Our findings imply that the promotion of pickled vegetable and fermented bean curd consumption could help to prevent diabetes, possibly by mitigating gut dysbiosis. However, further community-based interventional studies are needed to determine the efficacy of these dietary habits for preventing diabetes.

## Data availability statement

The raw data supporting the conclusions of this article will be made available by the authors, without undue reservation.

## Ethics statement

The studies involving human participants were reviewed and approved by Institutional Review Committee of Guizhou Center for Disease Control and Prevention. The patients/participants provided their written informed consent to participate in this study.

## Author contributions

YC and XY collected specimens, analyzed the data, and wrote the manuscript. TL, MC, and KT collected specimens and analyzed the data. SC, RD, YC, and KT analyzed the data. SX, TL, and KT contributed to the discussion. SX, TL, YY, and KT reviewed the manuscript. All authors contributed to the article and approved the submitted version.
