# Supplementary material for: Regular consumption of pickled vegetables and fermented bean curd reduces the risk of diabetes: a prospective cohort study
Source: Front Public Health. 2023 Apr 27;11:1155989. doi: 10.3389/fpubh.2023.1155989 (PMC10173413; doi:10.3389/fpubh.2023.1155989)
Supplement: Supplementary file 1 [file Table_1.docx]

Supplementary Material

Regular Consumption of Pickled Vegetables and Fermented Bean Curds Reduce Diabetes Risk: A Prospective Cohort Study

Yulan Cai^1, 2†^, Xiaoxia Yang^3†^, Siju Chen^3†^, Kunming Tian^3,4^, Suowen Xu^4^, Renli Deng^5^, Min Chen^6^, Yan Yang^1, 2*^, Tao Liu^6*^

**Correspondence:** Tao Liu, [liutao9099No_2@163.com](mailto:liutao9099No_2@163.com);

Yan Yang, [2006yangyan80@163.com](mailto:2006yangyan80@163.com)

^†^These authors contributed equally to this work and share first authorship.

**Supplemental Table 1. Adjusted Odds Ratios for Associations of Diabetes and Pickled Vegetables/Fermented Bean Curd Intake (Pickled Vegetables Intake and Fermented Bean Curd Intake were regard as Continuous Variable)**

| **Continuous Variable** | **Odds Ratio (95%Cl)** | | |  |
| --- | --- | --- | --- | --- |
|  | **Model 1** | **Model 2** | **Model 3** |  |
| Pickled vegetables intake (kg/month) | 0.38 (0.26, 0.58) | 0.42 (0.28, 0.63) | 0.39 (0.26, 0.60) |  |
| ***P****-*Value | <0.001 | | |  |
| Fermented bean curd intake (kg/month) | 0.17 (0.08, 0.36) | 0.18 (0.09, 0.39) | 0.17 (0.08, 0.36) |  |
| ***P****-*Value | <0.001 | | |  |

Model1: Unadjusted

Model2: Adjusted for age, sex, area, education, marital status.

Model3: Adjusted for model2+ exercise, alcohol intake, BMI, smoke, vegetable intake, meat intake, rice intake, hypertension, dyslipidemia.

**Supplemental Table 2. Adjusted Odds Ratios for Associations of Diabetes and Pickled Vegetables/Fermented Bean Curd Intake by excluding Subjects with the Occurrence of Diabetes within 2 Years of Follow-up**

| **Classified Variable** | **Odds Ratio (95% CI)** | | |
| --- | --- | --- | --- |
|  | **Model 1** | **Model 2** | **Model 3** |
| Pickled vegetables intake (kg/month) | | | |
| 0 | 1 [reference] | 1 [reference] | 1 [reference] |
| 0~0.5 | 0.74 (0.60, 0.90) | 0.77 (0.63, 0.94) | 0.75 (0.61, 0.93) |
| 0.5~ | 0.35 (0.22, 0.57) | 0.37 (0.23, 0.61) | 0.35 (0.22, 0.59) |
| ***P***-trend | <0.001 | | |
| Fermented bean curd intake | | |  |
| No | 1 [reference] | 1 [reference] | 1 [reference] |
| Yes | 0.65 (0.53, 0.80) | 0.68 (0.55, 0.85) | 0.67 (0.54, 0.83) |

Model1: Unadjusted

Model2: Adjusted for age, sex, area, education, marital status.

Model3: Adjusted for model2 + exercise, alcohol intake, BMI, smoke, vegetable intake, meat intake, rice intake, hypertension, dyslipidemia.

**Supplemental Table 3. Adjusted Odds Ratios for Associations of Diabetes and Pickled Vegetables/Fermented Bean Curd Intake by including Subjects with Abnormal Dietary Intake (Pickled Vegetables Intake and Fermented Bean Curd Intake were regard as Continuous Variable)**

| **Continuous Variable** | **Odds Ratio (95%Cl)** | | |
| --- | --- | --- | --- |
|  | **Model 1** | **Model 2** | **Model 3** |
| Pickled vegetables intake (kg/month) | 0.78 (0.64, 0.96) | 0.82 (0.67, 1.00) | 0.80 (0.65, 0.98) |
| ***P****-*Value | 0.017 | 0.045 | 0.029 |
| Fermented bean curd intake (kg/month) | 0.36 (0.22, 0.58) | 0.39 (0.24, 0.63) | 0.38 (0.23, 0.63) |
| ***P****-*Value | <0.001 | <0.001 | <0.001 |

Model1: Unadjusted

Model2: Adjusted for age, sex, area, education, marital status.

Model3: Adjusted for model2+ exercise, alcohol intake, BMI, smoke, vegetable intake, meat intake, rice intake, hypertension, dyslipidemia.

**Supplemental Table 4. Adjusted Odds Ratios for Associations of Diabetes and Pickled Vegetables/Fermented Bean Curd Intake by including Subjects with Abnormal Dietary Intake (Pickled Vegetables Intake and Fermented Bean Curd Intake were regard as Classified Variable)**

| **Classified Variable** | **Odds Ratio (95%Cl)** | | |
| --- | --- | --- | --- |
|  | **Model 1** | **Model 2** | **Model 3** |
| Pickled vegetables intake (kg/month) |  |  |  |
| 0 | 1 [reference] | 1 [reference] | 1 [reference] |
| 0~0.5 | 0.76(0.63, 0.92) | 0.79 (0.65, 0.97) | 0.77 (0.63, 0.94) |
| 0.5~ | 0.59(0.43, 0.81) | 0.63 (0.45, 0.87) | 0.60 (0.43, 0.83) |
| ***P***-trend | <0.001 | | |
| Fermented bean curd intake |  |  |  |
| No | 1 [reference] | 1 [reference] | 1 [reference] |
| Yes | 0.68(0.56, 0.83) | 0.72(0.59, 0.87) | 0.70(0.57, 0.86) |

Model1: Unadjusted

Model2: Adjusted for age, sex, area, education, marital status.

Model3: Adjusted for model2+ exercise, alcohol intake, BMI, smoke, vegetable intake, meat intake, rice intake, hypertension, dyslipidemia.
